# Successful Surgical Removal of the Largest Known Spleen

**DOI:** 10.1155/2020/6790808

**Published:** 2020-02-25

**Authors:** David J. Hall, Nam H. Dang, Christiana M. Shaw

**Affiliations:** ^1^Department of Surgery, University of Florida College of Medicine, USA; ^2^Division of Hematology & Oncology, Department of Medicine, University of Florida College of Medicine, USA

## Abstract

**Background:**

Splenic marginal zone lymphoma (SMZL) is a rare subtype of non-Hodgkin lymphoma that typically presents with symptomatic splenomegaly. The optimal treatment of SMZL not well established. *Case Presentation.* A 44-year-old man with a history of low-grade B-cell lymphoproliferative disorder previously treated with chemotherapy presented with a three-month history of rapidly enlarging abdominal girth. He was found to have large symptomatic splenomegaly by computed tomography. After workup, he underwent preoperative angioembolization of the splenic artery followed by successful splenectomy. The spleen measured 45 cm in greatest dimension and weighed 12.14 kg, more than 12% of the patient's total body weight, making this both the largest spleen on record as well as the largest spleen ever surgically removed. Pathology was consistent with splenic marginal zone lymphoma. The patient did well and was discharged home on postoperative day 3.

**Conclusions:**

SMZL is an infrequent condition that leads to progressive symptomatic splenomegaly which can be managed surgically providing symptomatic improvement and an overall satisfactory oncologic outcome. A multidisciplinary approach to complex cases of SMZL splenomegaly is imperative to achieving optimal outcomes.

## 1. Introduction

Splenic marginal zone lymphoma (SMZL) is a rare subtype of non-Hodgkin lymphoma that typically presents with symptomatic splenomegaly. The optimal treatment of SMZL is not well established and is frequently determined by the patient's symptoms and overall disease burden. We herein present the case of a patient with an oncologic history who developed rapidly progressive and symptomatic massive splenomegaly and underwent successful treatment with a multidisciplinary approach resulting in the successful surgical removal of the largest known spleen.

## 2. Case Presentation

**Figure 1:**
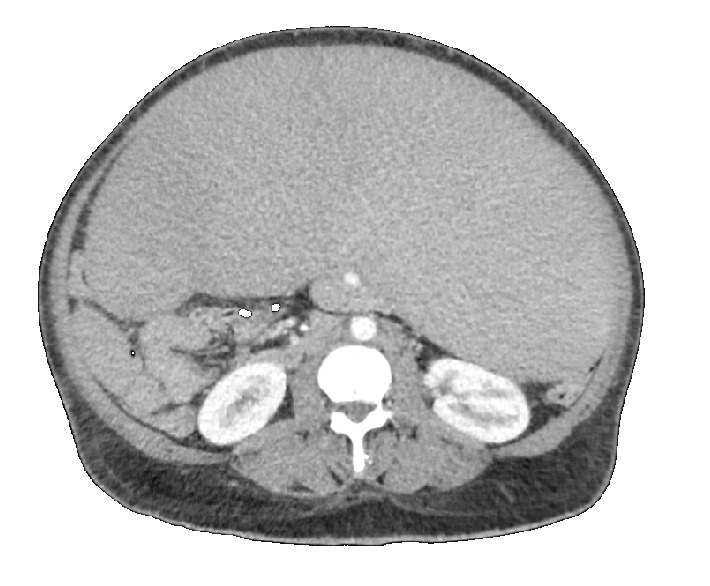
Preoperative computed tomography scan demonstrating marked splenomegaly.

A 44-year-old man with an unclear history of reported low-grade B-cell lymphoproliferative disorder previously treated with systemic chemotherapy at an outside facility greater than ten years ago presented with a three-month history of rapidly enlarging abdominal girth. He reported worsening nausea and early satiety, but was otherwise without fevers, chills, night sweats, or other complaints. Laboratory evaluation was significant for anemia (hemoglobin 10.0 g/dL), thrombocytopenia (72,000/mm^3^), and a leukocytosis (15,800/mm^3^). Bone marrow aspiration and peripheral blood flow studies demonstrated hypercellular bone marrow involving low-grade B-cell lymphoproliferative disorder consistent with marginal zone lymphoma with no evidence of large cell transformation. Computed tomography demonstrated massive splenomegaly with multiple mildly enlarged mediastinal and periaortic lymph nodes (Figure 1).

**Figure 2:**
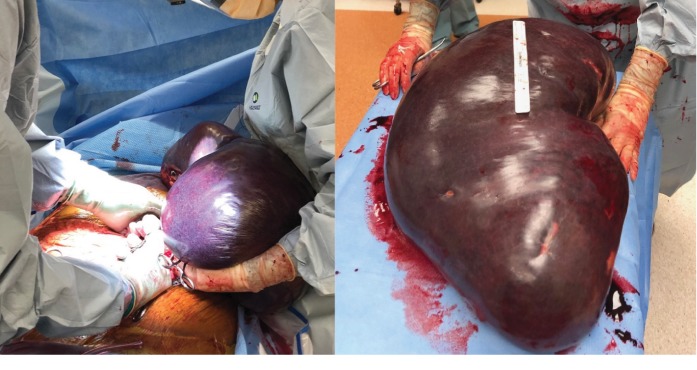
Intraoperative photographs demonstrating massive splenomegaly with successful splenectomy.

After discussion amongst the multidisciplinary team and the patient, the decision was made to proceed with splenectomy. He received preoperative splenic vaccines in the surgery clinic two weeks prior. On the morning of the surgery, he underwent splenic artery angioembolization followed by successful open splenectomy (Figure 2). Intraoperatively following the removal of the spleen, the patient experienced a drastic increase in blood pressure, which was managed medically and improved by the end of the procedure. The spleen measured 45 cm in greatest dimension and weighed 12.14 kg, more than 12% of the patient's total body weight, making this both the largest spleen on record as well as the largest spleen ever surgically removed. Pathology was consistent with splenic marginal zone lymphoma. The patient did well and was discharged home on postoperative day 3 and is undergoing surveillance by his medical oncologist prior to consideration of initiation of chemotherapy. This case demonstrates a successful multidisciplinary approach to splenic marginal zone lymphoma manifesting as massive splenomegaly.

## 3. Discussion

The patient described herein presented with rapidly progressing symptomatic abdominal enlargement that was ultimately identified on imaging to be the result of massive splenomegaly with peripheral blood and bone marrow samples consistent with marginal zone lymphoma, who then under the care of a multidisciplinary team underwent successful splenectomy with equally rapid improvement in his preoperative symptoms. This case is unusual in several aspects. Of importance, in cases of rapidly progressive lymphoma-related splenomegaly, there is typically histologic evidence of transformation into diffuse large B-cell lymphoma. This patient's preoperative and final pathologic specimen revealed only SMZL without large cell transformation. Secondly, we demonstrate a successful multidisciplinary approach to massive splenomegaly in a patient with SMZL involving preoperative evaluation by the medical oncology team, same-day angioembolization of the splenic artery, and splenectomy with intraoperative hemodynamic monitoring and optimization by the anesthesiology group. Finally, this case is significant in that it is the largest known spleen on record, and the largest spleen ever surgically removed on record.

Asymptomatic patients without splenomegaly or cytopenias may initially be managed with observation. Patients who develop nausea, abdominal discomfort, or splenomegaly-induced cytopenias require therapy, with options including surgery, chemotherapy, or radiation. For symptomatic patients with a suitable risk profile, surgery continues to be the mainstay of therapy. Splenectomy provides rapid symptomatic improvement as well as correction of cytopenias related to hypersplenism. In patients with SMZL who underwent splenectomy in a large series, the median progression-free survival was 8.25 years with 84% and 67% overall survival at 5 and 10 years, respectively [1]. Additionally, splenectomy has been shown to be relatively safe. A 2019 review by Fallah and Olszewski of 6450 patients with splenic lymphoma in the National Cancer Data Base from 2004 to 2013 found that 58% of patients were treated with splenectomy, with a thirty-day overall mortality of 4% [2].

While splenectomy has traditionally been the treatment of choice for symptomatic patients who are otherwise adequate surgical candidates, this frequently results in only a partial response due to persistent extrasplenic disease with nearly all patients having bone marrow involvement and warranting further therapy. Various chemotherapeutic strategies, including hepatitis C eradication, are frequently utilized for the treatment of SMZL and are reviewed elsewhere [3]. Immunotherapy has shown promise in offering potential effective treatment. Rituximab has shown promise as a first-line treatment for SMZL, improving complete response and disease-free survival rates alone or in combination with chemotherapy compared to chemotherapy alone in small series and is proposed as a replacement for splenectomy as first-line therapy [4, 5].

In patients who are refractory to or unsuitable for surgery or chemotherapy, splenic irradiation is an option, and additionally has the potential to amplify response rates to immunochemotherapy with Rituximab if the patient requires further treatments [6]. Splenic irradiation has been shown in several small case series to be effective in the palliation of pain and abdominal discomfort in splenomegaly with success rates between 50 and 91% and minimal side effects [7–9].

Due to its low overall incidence, the optimal therapy for SMZL remains controversial and is a topic of ongoing study. In an attempt to provide guidance for stratification and selection of risk-tailored treatment approaches, an international group reviewed 593 SMZL patients and developed criteria for the diagnosis, treatment initiation, response assessment, management, and prognostic stratification of SMZL patients [10]. Broader awareness of SMZL among physicians and practitioners will promote prompt referral for appropriate therapy and tailoring of a multidisciplinary approach.

SMZL is an infrequent condition, but leads to rapidly progressive symptomatic splenomegaly. We demonstrate that even cases of massive splenomegaly can be managed surgically providing equally rapid symptomatic improvement and an overall satisfactory oncologic outcome. A multidisciplinary approach to complex cases of SMZL splenomegaly is imperative to achieving optimal outcomes.

## Abbreviations

SMZL: Splenic marginal zone lymphoma.
